# Prediction of hybrid performance in maize with a ridge regression model employed to DNA markers and mRNA transcription profiles

**DOI:** 10.1186/s12864-016-2580-y

**Published:** 2016-03-29

**Authors:** Carola Zenke-Philippi, Alexander Thiemann, Felix Seifert, Tobias Schrag, Albrecht E. Melchinger, Stefan Scholten, Matthias Frisch

**Affiliations:** Institute of Agronomy and Plant Breeding II, Justus Liebig University, Giessen, 35392 Germany; Biocenter Klein Flottbek, Developmental Biology and Biotechnology, University of Hamburg, Hamburg, 22609 Germany; Institute of Plant Breeding, Seed Science, and Population Genetics, University of Hohenheim, Stuttgart, 70593 Germany

## Abstract

**Background:**

Ridge regression models can be used for predicting heterosis and hybrid performance. Their application to mRNA transcription profiles has not yet been investigated. Our objective was to compare the prediction accuracy of models employing mRNA transcription profiles with that of models employing genome-wide markers using a data set of 98 maize hybrids from a breeding program.

**Results:**

We predicted hybrid performance and mid-parent heterosis for grain yield and grain dry matter content and employed cross validation to assess the prediction accuracy. Prediction with a ridge regression model using random effects for mRNA transcription profiles resulted in similar prediction accuracies than employing the model to DNA markers. For hybrids, of which none of the parental inbred lines was part of the training set, the ridge regression model did not reach the prediction accuracy that was obtained with a model using transcriptome-based distances.

**Conclusion:**

We conclude that mRNA transcription profiles are a promising alternative to DNA markers for hybrid prediction, but further studies with larger data sets are required to investigate the superiority of alternative prediction models.

## Background

The resources for field trials in a hybrid breeding program are restricted and only a fraction of all possible hybrids that could potentially be generated by crossing the inbred lines developed in each cycle of the breeding program can be phenotypically evaluated. The principle of hybrid prediction is to link the performance of phenotypically evaluated hybrids to predictors, such as DNA markers or mRNA transcription profiles, that can be assessed in the parental lines of the hybrids. For each state of the predictor, its effect on the phenotype is estimated and these effects are then used to predict the performance of new hybrids.

DNA markers were employed for hybrid prediction in maize and proved to be superior to prediction approaches based solely on pedigree and phenotypic data [1–5]. First results on using the mRNA transcriptome for hybrid prediction with distance-based approaches [6] or regression-based approaches [7] showed promising results. Genome-wide prediction of general combining ability (GCA) or testcross performance [8–10] can be regarded as a special case of hybrid prediction where one parental component (the tester) is known and the effects of the predictors assessed at the second parental component are used for hybrid prediction. In this context, first results of using metabolites as predictors were successful [10] but showed a lower prediction accuracy than using SNP markers as predictors.

Two important situations can be distinguished in hybrid prediction. The first is that the parental lines of a potential hybrid have already been evaluated for testcross performance with other lines of the breeding pool. If such testcross data are available for both parental lines but the hybrid itself is not yet generated, then we refer to the hybrid as type 2 hybrid (testcross data for two parents available). The second situation is that the parental lines are entirely new and have not yet been evaluated in any test cross. Such hybrids are referred to as type 0 hybrids (testcross data for none of the parents available). The application of ridge regression models in combination with mRNA transcription profiles for the prediction of type 0 and type 2 hybrids has not yet been investigated.

The goal of our study was to investigate the prediction of grain yield and grain dry matter content using field data of 98 maize hybrids and AFLP (amplified fragment length polymorphism) marker data as well as mRNA transcription profiles of their 21 parental lines. In particular, our objectives were to (1) assess the accuracy of predicting hybrid performance with a random effects model using mRNA transcription profiles, (2) investigate the number of mRNA transcripts that are required for precise hybrid prediction, (3) compare the prediction accuracy of a random model employing mRNA with the prediction accuracy obtained with AFLP markers as well as the prediction accuracy of previously published approaches, and (4) draw conclusions on possible application in breeding programs for prediction of hybrid performance and heterosis of type 2 and type 0 hybrids.

## Methods

### Field data

The field data were presented in detail by [11], where the factorial we used for the present study was referred to as Experiment 1. Here we give only a brief overview. Seven flint and 14 dent elite inbreds developed in the maize breeding program of the University of Hohenheim were used as parental inbreds for 98=7×14 factorial crosses between both groups of inbreds. The inbreds comprised eight dent lines with Iowa Stiff Stalk Synthetic background and six with Iodent background. Four flint lines had a European Flint background and three a Flint/Lancaster background.

The factorial crosses were evaluated in 2002 at six agroecologically diverse locations in Germany (Bad Krozingen, Eckartsweier, Hohenheim, Landau, Sunching, Vechta). The trials were evaluated in two-row plots using *α* designs with two to three replications. Hybrid performance for grain yield was assessed in Mg ha ^−1^ adjusted to 155 g kg ^−1^ grain moisture and for grain dry matter content in percent. The mean hybrid performance for grain yield was 11.72 Mg ha ^−1^ and for grain dry matter content 67.7 % with broad sense heritabilities of 0.80 (grain yield) and 0.91 (grain dry matter content). The GCA (general combining ability) and SCA (specific combining ability) variance components as well as their interactions with the locations were significantly different from zero (*α*=0.05) for both traits. The ratios of SCA:GCA variance components were 1.12 (grain yield) and 0.42 (grain dry matter content).

### AFLP marker data

The inbred lines were assayed for AFLP markers with 20 primer combinations as described in detail by [11]. After removing markers with more than 10 % missing values and a gene diversity smaller than 0.2 the number of 970 high quality markers remained for the analysis.

### Gene expression data

Five seedlings of each of the 21 diverse dent and flint maize inbred lines were grown for seven days under controlled conditions (25 °C 16 h day, 21 °C 8 h night, 70 % air humidity). Whole seedling tissue of five biological replicates was frozen in liquid nitrogen, homogenized, and pooled before target labeling and hybridization. Total RNA was isolated, precipitated with LiCl (8M) and purified with the “NucleoSpin RNA Clean-up Kit” (Macherey-Nagel, Düren, Germany) and used to synthesize aminoallyl-labeled RNA (aaRNA) following the “Amino Allyl MessageAmp aRNA” System protocol (Applied Biosystems/Ambion, Austin, USA). aaRNA was coupled with fluorescence dyes Cy3 or Cy5 (GE Healthcare, Chalfont St. Giles, UK) and purified with RNeasy MinElute Kit (Qiagen, Hilden, Germany). The 46k array from the maize oligonucleotide array project [12], GEO platform GPL6438 was hybridized according to the manufacturer instructions. The micro-arrays were scanned (AppliedPrecision ArrayWorx Scanner, Applied Precision Inc., USA) and data was evaluated using GenePix Pro 4.0 (Molecular Devices, Sunnyvale, USA). For the micro-array experiment, an interwoven loop design [13] was applied. It resulted in 63 hybridizations of dent and flint lines by sampling each dent line five times and each flint line eight times.

For experimental validation of the micro-array experiment, two genes in eight different lines were evaluated by Quantitative RT-PCR, essentially in accordance with the micro-array data. For the validation of micro-array expression pattern copy DNA from total RNA of the inbred lines S028, F047, L024, S058, S044, PO33, L043, and F039 was produced with Superscript II (Thermo Fisher Scientific) according to the manufacturer’s protocol. Quantitative RT-PCR was conducted for the genes GRMZM2G057829, GRMZM2G021406 and the actin gene (accession number JO1238) with the primer pairs 5-‘GAAACCATAACAGACGCGTCATCACATC-3‘/5‘-CAGCAGGAGCAGAAGAGGGAAAAG-3‘, 5‘-TAGGCTGCTATTTGGGCACTTAGTTTTAC-3‘/5‘-CCAGTACGGGAGACATGTAGAGTTC-3‘, and 5‘-TCCTGACACTGAAGTACCCGATTGA-3‘/5‘-CGTTGTAGAAGGTGTGATGCCAGTT-3‘, respectively, with the iCycler iQ (BIORAD, Germany) and the qPCR MasterMix Plus for SYBR Green I (Reference: RT-SN2X- 03 + NRFL, Eurogentec, Seraing, Belgium) in triplicates. Actin expression values were used for data normalization before relative expression levels between lines were calculated. The micro-array data have been deposited in Gene Expression Omnibus (GEO) under the series accession GSE17754.

The gene-oriented probes together with spike-in probes were tested for statistically significant differential expression across all comparisons with a moderated F-test and subsequently with a nested F-test for each comparison of parental lines. The *limma* package [14] was applied for the tests. A false discovery rate [15] of 0.01 for all genes showing a fold change of at least 1.3 and log-2 expression intensity of at least 8 was used to detect significant differential expression between inbred lines [16]. In total, 10,810 genes were differentially expressed in at least one pair of parental lines of the factorial crosses. We refer to this set of predictors as ‘mRNA10k’, random samples of 1000 out of the 10,810 genes are referred to as ‘mRNAr1k’.

### Prediction model

To estimate the predictor effects, we used a linear model that relates the phenotype of a hybrid to the marker genotype or mRNA transcription profiles that were observed in the two parental lines of the hybrid:

(1) $$ \mathbf{y} = \mathbf{1} \beta_{0} + \mathbf{F} \mathbf{u} + \mathbf{M} \mathbf{v} + \mathbf{e}  $$

$$u_{j} \sim \mathrm{N} \left(0,{\sigma^{2}_{f}}\right) \quad\quad v_{j} \sim \mathrm{N} \left(0,{\sigma^{2}_{m}}\right) \quad\quad e_{i} \sim \mathrm{N} \left(0,{\sigma^{2}_{e}}\right) $$

**y** is the response vector consisting of the hybrid performance of the *i*=1…*n* hybrids, **1** is a vector of 1’s, and *β*_0_ a fixed intercept. **u** and **v** are the vectors of the genetic effects of the *j*=1…*p* predictors in the female and male parent, respectively. The design matrices **F** and **M** consist of values *f*_*i,j*_ and *m*_*i,j*_ that code the observation of the *j*th predictor at the *i*th hybrid. For marker data, *f*_*i,j*_ or *m*_*i,j*_ is 1 if the AFLP band was observed in a parent and 0 otherwise. For mRNA, the design matrices contain the gene expression of gene *j* in the parents of the *i*th hybrid, the columns of the design matrices **F** and **M** were normalized. For **F** the normalization was carried out according to

(2) $$ f_{i,j} = \frac{o_{i,j}} {\max\limits_{k \in \left\{ 1 \dots s\right\}} \left(o_{k,j} \right) }  $$

where *o*_*i,j*_ are non-normalized original values for gene expression, and *s* is the number of parental lines used as female parents. For **M** the normalization was carried out analogously.

The variances ${\hat {\sigma }^{2}_{f}}$, ${\hat {\sigma }^{2}_{m}}$, and ${\hat {\sigma }^{2}_{e}}$ were estimated by restricted maximum likelihood (REML). Then the effects $ \hat {\mathbf {u}}$ and $\hat {\mathbf {v}}$ were obtained by solving the mixed model equations [17].

With this model the genotypic value of hybrids can be predicted as

(3) $$ \hat{\mathbf{y}}^{\star} = \mathbf{1} \mu + \mathbf{F}^{\star} \hat{\mathbf{u}} + \mathbf{M}^{\star} \hat{\mathbf{v}}  $$

where **F**^⋆^ and **M**^⋆^ are the design matrices for the predictors observed at the parental lines of the hybrid. The GCA of inbred lines can be predicted as

(4) $$ \hat{\mathbf{g}}_{f}^{\star} = \mathbf{F}^{\star} \hat{\mathbf{u}} \quad\quad\text{or}\quad\quad \hat{\mathbf{g}}_{m}^{\star} = \mathbf{M}^{\star} \hat{\mathbf{v}}   $$

### Assessment of prediction accuracy

**Fig. 1:**
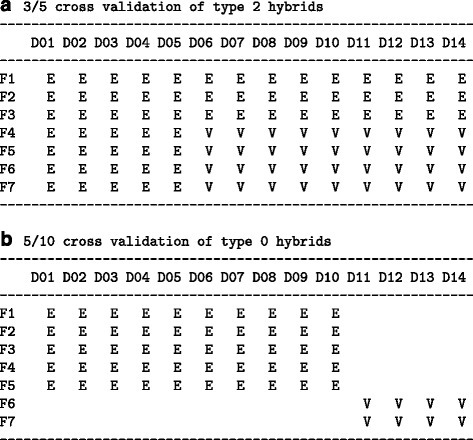
Cross validation schemes. **a** Evaluation of prediction accuracy for untested hybrids in an incomplete factorial. The hybrids in the validation set are of type 2. **b** Evaluation of prediction accuracy for hybrids derived from parental lines of which no testcross data are available. The hybrids in the validation set are of type 0. D01–D14: parental dent lines in random order, F01–F07: parental flint lines in random order, E: hybrids of the estimation set, V: hybrids of the validation set

The prediction accuracy for type 2 hybrids was evaluated with the cross-validation procedure of [3]. The estimation set consisted of the marker or mRNA data of three randomly chosen flint and five randomly chosen dent lines and the field data of their hybrids, and the validation set consisted of the remaining hybrids of the 7×14 factorial. Both parental lines of an untested hybrid in the validation set are also parents of hybrids belonging to the estimation set. Hence, testcross data are available for both parental lines of a hybrid. The principle is illustrated in Fig. 1a.

For type 0 hybrids, the estimation set consisted of five randomly chosen flint lines and ten randomly chosen dent lines and their hybrids. The validation set consisted of the hybrids of the remaining two flint and four dent lines of the 7×14 factorial. Hence, testcross data were not available for any of the two parental lines of a hybrid (Fig. 1b).

For each prediction model to be evaluated, cross-validation was carried out for 1000 runs. In each run the correlation $r(y,\hat {y})$ between the predicted and the observed hybrid yield and the average prediction error $\sum |\hat {y}_{i}- y_{i}| / n$ was assessed. The distribution of these measures over the 1000 replications was then used to compare the prediction models.

## Results

**Fig. 2:**
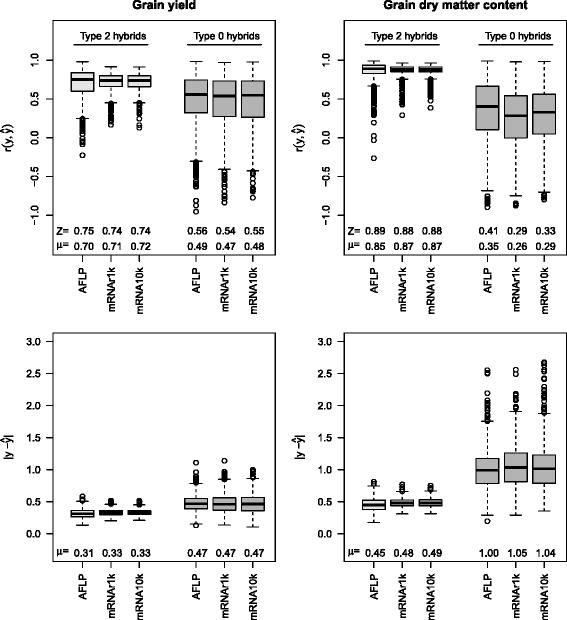
Prediction accuracy for hybrid performance of type 2 hybrids (*left, in light gray*) and type 0 hybrids (*right, in dark gray*). Correlations $r(y,\hat {y})$ between observed and predicted grain yield and grain dry matter content, and average absolute prediction error $|y-\hat {y}|$ for the predictor sets AFLP (970 AFLP markers), mRNAr1k (1000 random mRNA transcripts), mRNA10k (10,810 mRNA transcripts). The boxplots show the distributions for 1000 cross validation runs, *μ* are the arithmetic means and *Z* the medians

For prediction of hybrid performance, the median of the correlations $r(y,\hat {y})$ between observed and predicted values in cross validation with type 2 hybrids was between 0.74 and 0.75 for grain yield and between 0.88 and 0.99 for grain dry matter content (Fig. 2). The differences in the median of the correlation between prediction with AFLPs, with all 10k mRNAs (mRNA10k), and with random samples of 1k out of the 10k mRNAs (mRNAr1k) were negligible. Prediction with mRNAs had a slightly smaller variation around the median than prediction with AFLPs. The average absolute prediction errors $|y-\hat {y}|$ had about the same sizes for prediction with AFLPs, all 10k mRNAs and random samples of 1k out of the 10k mRNAs.

For type 0 hybrids, the correlations between observed and predicted hybrid performance for both traits were lower than for type 2 hybrids. The median of the correlations in cross validation was between 0.54 and 0.56 for grain yield and between 0.29 and 0.41 for grain dry matter content. Differences in the median between the predictor sets AFLP, mRNA10k, and mRNAr1k were small. The ranges of the correlations were very large, and in some cross validation runs, even large negative correlations were observed. The average absolute prediction errors were greater than for type 2 hybrids and showed similar values for AFLPs and mRNA.

**Fig. 3:**
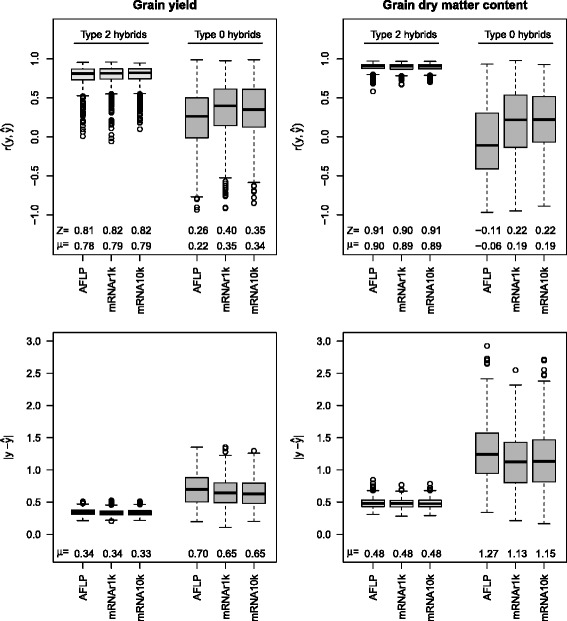
Prediction accuracy for mid-parent heterosis of type 2 hybrids (*left, in light gray*) and type 0 hybrids (*right, in dark gray*). Correlations $r(y,\hat {y})$ between observed and predicted grain yield and grain dry matter content, and average absolute prediction error $|y-\hat {y}|$ for the predictor sets AFLP (970 AFLP markers), mRNAr1k (1000 random mRNA transcripts), mRNA10k (10,810 mRNA transcripts). The boxplots show the distributions for 1000 cross validation runs, *μ* are the arithmetic means and *Z* the medians

For prediction of mid-parent heterosis, the median of $r(y,\hat {y})$ with type 2 hybrids was between 0.81 and 0.82 for grain yield and between 0.90 and 0.91 for grain dry matter content (Fig. 3). The differences between the predictor sets AFLP, mRNA10k, mRNAr1k were negligible. The average absolute prediction error $|y-\hat {y}|$ had about the same sizes for the three predictor sets.

For type 0 hybrids, the correlations between observed and predicted mid-parent heterosis were between 0.26 and 0.4 for grain yield. For grain dry matter content no correlation between observed and predicted values in cross validation was observed.

In additional analyses we investigated the effect of further reducing the number of predictor variables below 1000. A decline of the prediction accuracy was observed for both traits (results not shown), which is in line with the results of [6].

We further investigated a ridge regression model in which we included 1000 random mRNAs and in addition the AFLP markers as predictors. We found no situation where combining the predictor sets resulted in a greater prediction accuracy than using them individually (results not shown).

## Discussion

### Properties of the linear model

In a simple GCA/SCA model *y*_*fmr*_=*μ*+*g*_*f*_+*g*_*m*_+*s*_*fm*_+*e*_*fmr*_ the performance of the *r*th replication of a hybrid is denoted by *y*_*fmr*_. Factors *g*_*f*_ and *g*_*m*_ describe the GCA values of the parental lines, and *s*_*fm*_ is the SCA of the cross. In the linear model of Eq. 1, the GCA values are split into components that can be assigned to individual predictors, **Fv** splits up *g*_*f*_ and **Mu** splits up *g*_*m*_.

Heterosis, and in consequence high hybrid performance, can be explained by dominant gene action at a large number of loci. Therefore, it is essential that models that attempt to predict hybrid performance include the effect of dominant gene action. The *u*_*j*_ and *v*_*j*_ in Eq. 1 can be interpreted in the sense of average effects (using the terminology of [18] p. 112ff) of the corresponding predictors. Average effects cover the effect *a* of additive gene action, and in addition they partially cover the effect *d* of dominant gene action (cf. Eq. 7.4a and 7.4b of [18], p. 113). The amount of the dominant gene action that is captured depends on the differences in the allele frequencies, and takes its minimum of zero for allele frequencies of 1/2. We hypothesize, that the differences in the allele frequencies in the heterotic pools of our factorial are so large that the average values include to a large extend the effect of dominant gene action. This is supported by the high prediction accuracies observed.

The SCA is neglected in Eq. 1. Extensions that include the SCA are straightforward from a formal point of view (Eq. 4 in [4]). The dissection of the SCA into components that can be assigned to individual predictors results in effects that can be interpreted in the sense of dominance deviations (cf. Table 7.3 of [18], p. 118). Dominance deviations cover the residual part of the effect of dominant gene action *d*, that is not covered by the average effects. Simulations have shown that the gain in prediction accuracy of models that include dominance deviations is small for divergent heterotic pools [4], because the major part of the effect of dominant gene action *d* is already covered by the average effects.

It remains open, and requires the analysis of further experimental data sets, whether including SCA in prediction models can actually improve hybrid prediction. In the data set investigated here, the high correlations of up to $r(y,\hat {y}) =0.9$ between observations and predictions leave only little room for improving the GCA-based approach.

### Prediction accuracy compared with older approaches

In earlier investigations on marker-based [11] and transcriptome-based [6, 7] prediction of hybrid performance, we used the same set of hybrids as here. This allows a direct comparison of the accuracy of the different prediction methods.

In the SM-TEAM approach of [11], first all markers are tested for association with the target trait and then a fixed linear model for the selected markers is fitted. This procedure is in analogy to the QTL-mapping approach, whereas a random model in which all markers remain (Eq. 1) can be regarded as a genome-wide prediction approach, as employed in recent studies on genomic selection. Hence, the theoretical advantages of the genome-wide prediction model, such as less bias in the effect estimates, should result in better statistical properties of the approach presented here compared with the approach of [11]. The correlation between predicted and observed hybrid performance for grain yield of type 2 hybrids obtained by the SM-TEAM approach was 0.65 (Figure 6 in [6]). The random effects model with AFLPs had a median of the correlation of 0.75 (Fig. 2). In consequence, with the present factorial, the ridge regression model applied to DNA marker data had a greater prediction accuracy than the earlier SM-TEAM model.

Transcriptome-based distances reached a prediction accuracy of about 0.8 for hybrid performance and mid parent heterosis of grain yield in type 2 hybrids (Figure 6 in [6]). This value is similar to the prediction accuracy reached by the ridge regression model (Fig. 3) for mid-parent heterosis. However, for hybrid performance, the ridge regression model showed only a correlation of 0.75 (Fig. 2), and, hence could not reach the prediction accuracies of the transcriptome-based distance model.

For prediction of hybrid performance for grain yield of type 0 hybrids, the transcriptome-based distances reached a median of the correlation between observations and predictions of 0.7 (Figure 3 in [7]). This was considerably greater than the regression-based methods investigated in [7]. For the ridge regression model, a median of the correlation of about 0.55 was reached (Fig. 2). In consequence, for the prediction of type 0 hybrids the transcriptome-based distance model, which employs marker selection, resulted in considerably better predictions than the ridge regression model of this study.

### Application in breeding programs

For application of hybrid prediction in breeding programs, it is of central importance that a prediction approach provides a sufficiently high prediction accuracy. For indirect selection approaches, a correlation of 0.7 to 0.9 between the trait for which selection is carried out and the target trait is usually regarded as highly promising and applicable in practice. Hence, the prediction accuracies for type 2 observed in this study can be regarded as suitable for practical applications.

The prediction accuracy of employing the ridge regression model to mRNAs was comparable to that obtained with AFLP markers in the investigated data set. The accuracies for prediction of grain yield and grain dry matter content in type 2 hybrids (Figs. 2 and 3) which were achieved with mRNA data suggest than mRNA can be an alternative to DNA markers in hybrid prediction.

The number of mRNAs required for a high prediction accuracy plays a central role for the costs of assessing the transcription profiles of selection candidates. For both traits and for both types of hybrids, the differences between using 1000 randomly chosen mRNAs or 10,000 mRNAs were negligible. This indicates, that high numbers of mRNA are not necessarily required for hybrid prediction, and that transcription profiling with limited resources might result in prediction accuracies that can be successfully used for indirect selection.

The ridge regression model employed in this study was in summary more precise than the older SM-TEAM prediction model. However it was not superior to the transcriptome based distances suggested by [6]. In particular for prediction of type 0 hybrids, the transcriptome-based distances might be the more promising approach. Further studies with larger data sets are required to verify these trends.

## Conclusions

Hybrid prediction has the potential to greatly enhance the efficiency of hybrid breeding. In maize breeding, the doubled haploid technology can generate large numbers of candidate lines that surpass the field capacity by far. Thus, reliable hybrid prediction can be used to increase the selection intensity and hence the response to selection. The data structure of the factorial used in this study is typical for testing experimental hybrids in late stages of a maize hybrid breeding program, and hence the successful application of hybrid prediction with mRNA and ridge regression prediction models can be also expected with other data sets of similar genetic structure.

## Abbreviations

AFLP: amplified fragment length polymorphism
GCA: general combining ability
SCA: specific combining ability
